# Metabolic rate measurements of two benthic invertebrates under simulated climate change conditions

**DOI:** 10.3897/BDJ.14.e187026

**Published:** 2026-03-17

**Authors:** Panagiotis Grigoriou, Eva Chatzinikolaou, Athanasios Anastasiadis, Nikos Papandroulakis, Orestis Stavrakidis-Zachou, Thanos Dailianis

**Affiliations:** 1 Hellenic Centre for Marine Research (HCMR), Cretaquarium, Heraklion, Crete, Greece Hellenic Centre for Marine Research (HCMR), Cretaquarium Heraklion, Crete Greece; 2 Hellenic Centre for Marine Research (HCMR), Institute of Marine Biology, Biotechnology and Aquaculture (IMBBC), Heraklion, Crete, Greece Hellenic Centre for Marine Research (HCMR), Institute of Marine Biology, Biotechnology and Aquaculture (IMBBC) Heraklion, Crete Greece

**Keywords:** oxygen consumption, respirometer, *Hexaplex trunculus*, *Chondrilla nucula*, gastropods, sponge, warming, ocean acidification, Mediterranean

## Abstract

**Background:**

Climate change is profoundly altering marine ecosystems through ocean warming and acidification. These stressors are especially pronounced in the Mediterranean Sea, a climate change hotspot projected to warm faster than the global average. Increased temperatures and reduced pH directly affect metabolic processes in marine invertebrates by elevating respiration rates up to species-specific thermal limits, beyond which physiological performance declines. Ocean acidification further disrupts metabolic processes by increasing energetic maintenance costs. Sessile and sedentary marine invertebrates, such as sponges and benthic gastropods, are particularly exposed to such environmental shifts due to their limited ability to escape unfavorable conditions, making physiological plasticity and local adaptation crucial for persistence.

**New information:**

This manuscript presents a dataset of oxygen consumption rates and wet weight measurements for two low-mobility marine species, the gastropod *Hexaplex trunculus* and the sponge *Chondrilla nucula*. Using a common garden experiment, individuals from North and South Aegean populations were exposed for three months to simulated climate change conditions combining increased temperature and reduced pH. The dataset documents respiration measurements obtained using metabolic chambers after three months of exposure, allowing comparisons across species, geographic origin, and experimental treatments.The dataset accounts for intraspecific variation in these responses, providing insight into potential adaptive differences among geographically distinct populations. These data provide a resource for future analyses of metabolic responses of marine invertebrates to combined warming and acidification conditions.

## Introduction

Climate change is a major driver of ongoing alterations in marine ecosystems, primarily through ocean warming and ocean acidification, both of which are directly linked to increased atmospheric CO₂ concentrations (Calvin et al. 2023). Sea surface temperatures have risen significantly over the past century, and future projections indicate continued warming, particularly in semi-enclosed basins such as the Mediterranean Sea, which is considered a climate change hotspot (Christou et al. 2025). In the Mediterranean region temperature will rise by 20% more than the global average during the twenty-first century (Lionello and Scarascia 2018). At the same time the absorption of anthropogenic CO₂ by seawater has resulted in a decrease in ocean pH, altering carbonate chemistry and posing additional challenges to marine organisms (Doney et al. 2009).

Temperature is a fundamental factor controlling metabolic processes in ectothermic organisms. In marine invertebrates, elevated temperatures generally lead to increased respiration rates due to accelerated enzymatic and biochemical reactions, as described by the metabolic theory (Gillooly et al. 2001). However, when temperatures exceed species-specific thermal optima, metabolic performance may decline, resulting in reduced aerobic scope, metabolic depression, and compromised physiological function (Pörtner and Farrell 2008). Ocean acidification further affects metabolism by disrupting acid–base balance, enzyme activity, and energy budgets, often increasing maintenance costs and reducing energy available for growth and reproduction (Leung et al. 2020, Melzner et al. 2009). Numerous experimental studies have demonstrated that the combined effects of increased temperature and reduced pH can interact in complex ways, producing synergistic or antagonistic impacts on respiration, calcification, and survival in marine invertebrates (Chatzinikolaou et al. 2016, Kroeker et al. 2013, Harvey et al. 2013).

Marine invertebrates with limited motility are particularly vulnerable to climate change–induced environmental stressors. Sessile organisms, such as sponges, and sedentary benthic mollusks are unable to avoid unfavorable conditions and therefore rely heavily on physiological plasticity or local adaptation to cope with environmental variability (Somero 2010). Sponges play a crucial role in benthic–pelagic coupling and nutrient cycling, and changes in their metabolic rates may have cascading effects on ecosystem functioning (Maggioni et al. 2023). Similarly, benthic gastropods such as *Hexaplex trunculus* are key components of coastal food webs and their metabolic sensitivity to warming and acidification may influence population dynamics and trophic interactions (Gazeau et al. 2013).

In recent years, increasing attention has been given to intraspecific variation as a critical determinant of species resilience to climate change. Populations inhabiting different geographic regions often experience distinct environmental regimes and may develop divergent physiological traits through local adaptation or long-term acclimatization (Kawecki and Ebert 2004, Sanford and Kelly 2011). Common garden experiments, in which individuals from different populations are exposed to identical environmental conditions, provide a powerful approach to investigate population-level differences in physiological performance and to identify potential adaptive diversity among geographically distinct populations (de Villemereuil et al. 2015).

The present data paper describes a dataset of respiration measurements obtained for two benthic marine invertebrates maintained under controlled combinations of temperature and pH. The dataset includes individuals originating from two distinct Aegean populations that were exposed to simulated climate change scenarios in a common garden experimental set-up.

### Aim of the project

The aim of this study was to investigate the impact of ocean warming and acidification on the metabolic responses of two species with partial or low motility, the marine gastropod *Hexaplex trunculus* (Mollusca: Gastropoda) and the sponge *Chondrilla nucula* (Porifera: Demospongiae). The proposed approach involves experimental exposure of the targeted organisms to climate change scenarios, in order to simulate the synergistic impact of naturally occurring stressors, more specifically ocean warming and acidification. Individuals collected from a North Aegean and a South Aegean population in Greece, were used in a common garden experiment in order to assess the responses of these organisms to thermal and oxidative stress, with the additional aim of investigating intraspecific variation in these responses and thereby uncovering potential adaptive diversity among geographically distinct populations.

## Project description

### Title

Multi-level Approaches to assess Climate Change Impact to Marine Organisms (MACCIMO)

### Personnel

Dr Thanos Dailianis (scientific responsible, experimental design), Dr Eva Chatzinikolaou (experimental design, WP2 leader), Dr Panagiotis Grigoriou (experimental design, technical support, WP4 leader), Athanasios Anastasiadis (technical support)

### Study area description

Crete - South Aegean (35.3357°N, 25.2815°S, up to 5 m depth) and Chalkidiki - North Aegean (39.9315°N, 23.7348°S, up to 5 m depth), Mediterranean Sea, Greece

### Design description

Three climate change scenarios were simulated during the experiments: 1) the Control scenario in which the ambient temperature was estimated as the average of the maximum summer temperatures (27°C) between the two populations (North and South Greece). The pH was ambient (~8.1); 2) the South Aegean Climate Change (SACC) scenario ("extreme") in which temperature was estimated as the maximum recorded in South Aegean (Crete) during summer (27°C) increased by 4°C. The pH was decreased by 0.3 units (~7.8); and 3) the North Aegean Climate Change (NACC) scenario ("mild") in which temperature was estimated as the maximum recorded in North Aegean (Chalkidiki) during summer (26°C) increased by 4°C. The pH was decreased by 0.3 units (~7.8).

The selected Climate Change scenarios (i.e. final temperature increase by 4°C and final pH decrease by 0.3 units) were based on the "high GHG emissions" RCP 8.5 scenario of IPCC as this is described in the Climate Change 2023: Synthesis Report (Calvin et al. 2023).

### Funding

This work was funded by the Hellenic Foundation for Research and Innovation (HFRI) under the “2nd Call for HFRI Research Projects to support Faculty Members & Researchers”, Project Number 3280.

## Sampling methods

### Sampling description

**Selection of species**

The sponge *Chondrilla nucula* Schmidt, 1862 and the gastropod *Hexaplex trunculus* (Linnaeus, 1758) were selected as the experimental organisms in this study. *C. nucula* is a photophilic sponge characterized by a modular growth form and clonal body masses that often spreads extensively on hard substrates (Milanese et al. 2003). It is a broadly distributed species occurring in diverse habitats, primarily in shallow coastal waters down to depths of about 30 m (Strano et al. 2020), but it has also been reported in deeper environments (Stamouli et al. 2023). *C. nucula* is a filter-feeder, actively circulating water and particulate matter through its body, thereby contributing significantly to nutrient cycling and the maintenance of water quality in marine ecosystems (Milanese et al. 2003). Its highly competitive ability for space often allows it to dominate certain benthic communities (Strano et al. 2020). *C. nucula* is of considerable commercial and biotechnological interest due to its production of bioactive compounds (Chelossi et al. 2007).

*Hexaplex trunculus* is a cosmopolitan species inhabiting both intertidal and subtidal zones, exhibiting a strong resilience and adaptability to fluctuating environmental conditions (Lahbib et al. 2010). Reproduction occurs with egg capsules, from which juveniles emerge through direct development, bypassing a planktonic larval stage (Lahbib et al. 2012). *H. trunculus* preys on both live and dead organisms, including bivalves and other gastropods (Peharda and Morton 2005) and can be readily maintained under laboratory conditions. It is an edible species and holds significant commercial value in several countries, such as Italy, Tunisia, Portugal, and Spain (Vasconcelos et al. 2006, Lahbib et al. 2012). Moreover, *H. trunculus* serves as a key ecological indicator of marine pollution by organotin compounds, including tributyltin (TBT) (Abidli et al. 2012).

**Experimental set-up**

The experimental set-up used to adjust the treatment tanks to the desired levels of temperature and pH, as well as the protocols for the maintenance of organisms during the 3 months of the experiment, have been described in detail in the first manuscript of the Special Issue series "Multi-level assessment of climate change impacts in benthic marine invertebrates: insights from the MACCIMO project", which is published by Chatzinikolaou et al. 2026.

**Collection of samples**

A group of individuals from each species and each population (north and south) per treatment were randomly selected for the respiration measurements (i.e. oxygen consumption rates) following the end of the experiment after 3 months. All organisms were not fed for 2 days prior to respiration measurements.

**Measurement of respiration rates in metabolic chambers**

**Figure 1.**
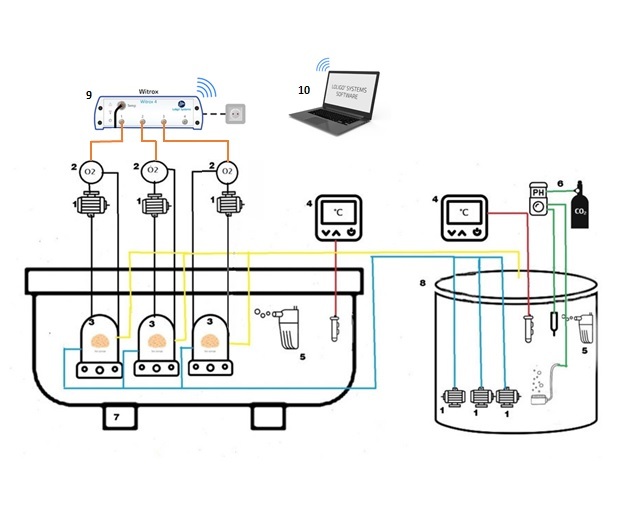
Experimental set-up for measuring respiration rate in marine invertebrates. **Red lines**: cables connecting temperature controller and heater. **Yellow lines**: pipes for removal of used water. **Blue lines**: pipes for inserting fresh water. **Green lines**: cables connecting pH controller with pH electrode and CO_2_ system. **Black lines**: pipes transferring water to the sensor sockets for O_2_ measurement. **Orange lines**: cables connecting oxygen sensors with Witrox 4 oxygen meter **1**: water pumps, **2**: sockets hosting optic oxygen sensors, **3**: respiration chambers, **4**: temperature controllers and titanium aquarium heaters, **5**: water pump, **6**: CO_2_ system including manometer, pH controller system, CO_2_ valve set and pH electrode, **7**: Water bath, **8**: Seawater providing tank, **9**: Witrox 4 oxygen meter and **10**: laptop receiving measurements.

**Figure 2.**
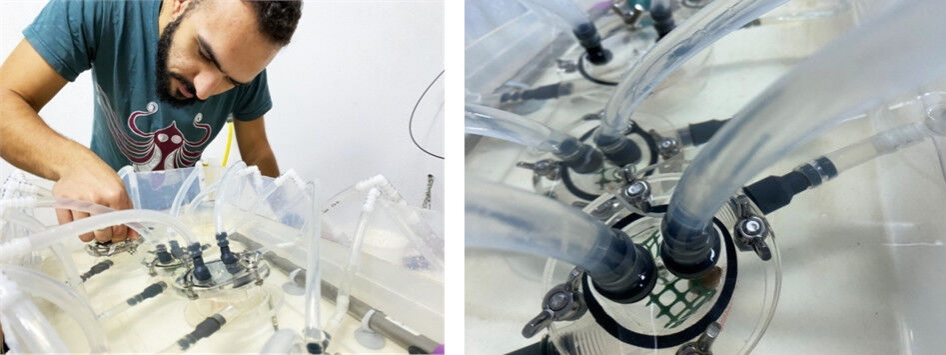
The respiration rate was measured inside individual chambers.

The experimental set-up for measuring respiration rate in marine invertebrates (sponges and gastropods) was developed as shown in Fig. 1 and Fig. 2. A “seawater providing tank” (40L) was set up (Fig. 1, right) which contained seawater adjusted at the temperature and pH conditions according to the experimental treatment tested each time. A water pump (NEW JET NJ600 aquarium pump) and an air pump (EHEIM air 100) were installed in this tank in order to achieve 100% oxygen saturation. Temperature was monitored and controlled according to the conditions of the respective experimental treatment using a D-D dual heating and cooling controller. A temperature sensor was submerged in the tank and connected to a submerged aquarium heater (D-D Titanium Heater) to raise the temperature to the desired level. The pH was monitored and controlled by a pH/CO_2_ controller (Tunze pH controller 7070/2 and CO_2_ valve set 7074.110). A CO_2_ bubbling system (CO_2_ art) was used to adjust pH at the desired level at the low pH treatments (SACC and NACC treatments), which was connected to a CO_2_ bottle (2L) via a manometer (Gloor) and to the controller system. The pH electrodes were calibrated once at the beginning of testing for each treatment due to the short time period of the respiration measurement experiments using Tunze buffer solutions for pH 7 (7040.117) and 9 (7040.110).

Three acrylic respiration chambers of 35 mL volume (Loligo systems) were used to measure the oxygen consumption rate of sponges and gastropods (Fig. 2). At the beginning of each respiration measurement, oxygen-saturated seawater from the “seawater providing tank” was added through water pumps (EHEIM universal 300, 5L/min) inside the respiration chambers, ensuring that the oxygen consumption rate was estimated under the respective experimental conditions. The pumps also ensured circulation and mixing of the seawater within the chambers to create sufficient water flow past the oxygen electrode. A pipe on the lid of the respiration chamber served to remove bubbles from the oxygen-saturated seawater after each water renewal. Oxygen was measured using a fiber optic cable which was inserted in a flow-through oxygen cell (i.e sockets hosting the Loligo mini sensors) within the recirculating loop and was connected to the Witrox 4 oxygen meter. The respiration rate was calculated as mgO_2_/g/h, where g refers to the wet weight of the organism. The chambers were submerged in a water bath (13L) (Fig. 1, left) to maintain a constant water temperature during the respiration measurements using a D-D Titanium Heater which was connected to the D-D Dual Heating & Cooling Controller. A water pump (NEW JET NJ600 aquarium pump) was also placed inside the water bath tank to ensure appropriate mixing of the water.

The respiration rate was calculated by the software Autoresp v2.3 taking into account the chamber volume (35ml), the total volume of the system tubes and the wet weight of the organism. The software then automatically calculated the effective respiration water volume. Initially each organism remained inside the chamber for 30 min to recover from handling before any measurements were made, since after that period the oxygen consumption rate was stabilized. Following this period, fresh seawater was introduced to the chamber and the oxygen tension inside the chamber was continuously logged to a computer. Each cycle consisted of three phases: the "flush" phase during which fresh water was introduced (180 seconds), the "wait" phase during which no values were recorded (120 seconds) and the "measurement" phase which had a duration of about 1200 seconds depending on animal size (in order to avoid oxygen drop bellow 80%). Respiration rates were measured under light conditions in individuals from each treatment and each population, while each individual was measured at least over four measuring cycles and the average value was calculated. Oxygen consumption slopes with R^2^ less than 90% were not acceptable and were not included in the estimation of mean values. Corrections (i.e. blanks) for electrode and micro-organism oxygen consumption were performed every 2 or 3 readings during each experimental temperature by measuring the oxygen consumption in the chamber without an organism present.

**Measuring respiration in sponges *Chondrilla nucula***

During maintenance in the experimental treatments, sponges *Chondrilla nucula* were placed individually in specially designed permeable enclosures made with plastic net to prevent mixing of populations and their escape from the experimental tanks. At the end of the 3-month experiment, part of this plastic net had been tightly surrounded by the sponge and its removal without injuring the organisms was not possible. Therefore, each sponge was individually placed in the respiration chamber attached to a small piece of the plastic net. The piece of plastic net also facilitated keeping individuals in a stable position inside the chamber and avoid their drifting during the respiration measurements. The wet weight of sponges (required as input in the respiration calculation software Autoresp v2.3) was measured after removing excess moisture with absorbent paper using a precision balance (0.001g). The piece of plastic net was negligible in comparison to the total effective respiration volume.

**Measuring respiration in *Hexaplex trunculus***

**Figure 3.**
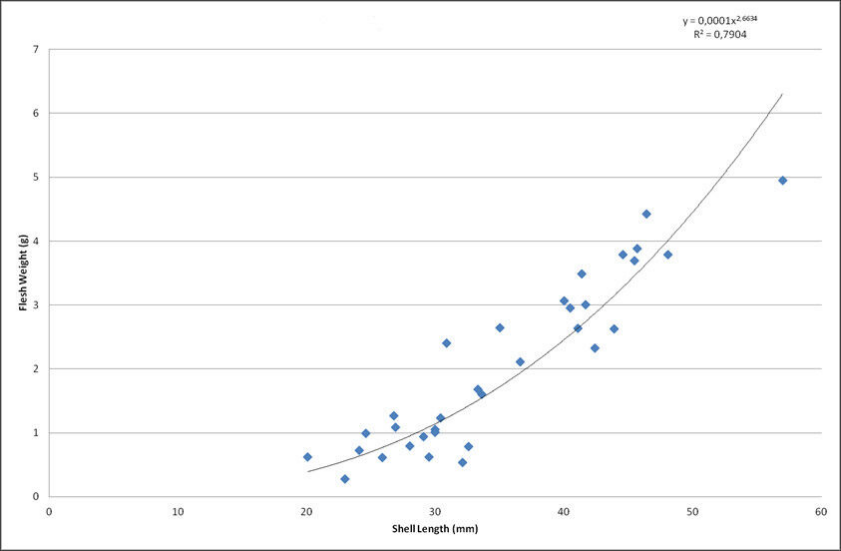
Relationship between shell length (mm) and wet weight (g) (excluding shell) for the gastropod *Hexaplex trunculus*.

Gastropods *Hexaplex trunculus* were placed individually in the respiration chambers after their shell length had been measured to the nearest 0.01 mm using a vernier caliper. Respiration rates were measured in individuals from each treatment (number depending on availability) and each population. The wet weight of gastropods (excluding shell) (required as input in the respiration calculation software Autoresp v2.3) was estimated from their shell length using a reference equation. Gastropods (n=33) of a range of different sizes (20-60 mm), similar to those in the experimental treatments, were used to calculate an equation between shell length (mm) and body wet weight (g) (excluding shell) (Fig. 3). Total wet weight was measured in a precision laboratory balance (0.001g) after blotting away the excess water. Then the specimens were anesthetized using a rising concentration of MgCl_2_ starting at 1.5% and gradually reaching 3.5% according to the European Directive 2010/63 EU on the protection of animals used for scientific purposes. The gastropods were cracked open and their shell was removed in order to measure body wet weight.

## Geographic coverage

### Description

South Aegean - Crete (35.3357/25.2815); North Aegean - Chalkidiki (39.9315/23.7348)

### Coordinates

35.3357 and 39.9315 Latitude; 23.7348 and 25.2815 Longitude.

## Taxonomic coverage

### Description

Phylum: Mollusca, Class: Gastropoda, Order: Neogastropoda, Family: Muricidae, Genus: Hexaplex, Species: *Hexaplex trunculus*

Phylum: Porifera, Class: Demospongiae, Order: Chondrillida, Family: Chondrillidae, Genus: Chondrilla, Species: *Chondrilla nucula*

### Taxa included

**Table:** 

Rank	Scientific Name	Common Name
species	*Hexaplex trunculus*	banded dye-murex
species	*Chondrilla nucula*	chicken-liver sponge

## Usage licence

### Usage licence

Other

### IP rights notes

The publisher and rights holder of this work is Hellenic Center for Marine Research. This work is licensed under a Creative Commons Attribution (CC-BY 4.0) License.

## Data resources

### Data package title

Oxygen consumption data of *Chondrilla nucula* and *Hexaplex trunculus* subjected to experimental climate change conditions

### Resource link

https://doi.org/10.25607/84dmh8

### Alternative identifiers

https://ipt.medobis.eu/resource?r=oxcons_chondrilla_hexaplex_climatechange; https://www.gbif.org/dataset/690aa1a7-3d0d-4c11-abcc-b4525deb1d1d

### Number of data sets

1

### Data set 1.

#### Data set name

Oxygen consumption data of *Chondrilla nucula* and *Hexaplex trunculus* subjected to experimental climate change conditions

#### Data format

Darwin Core Archive (DwC-A)

#### Download URL

https://ipt.medobis.eu/resource?r=oxcons_chondrilla_hexaplex_climatechange

#### Data format version

1.0

#### Description

This dataset includes respiration data for two invertebrate species, the sponge *Chondrilla nucula* and the gastropod *Hexaplex trunculus*, which were produced using an experimental set up with metabolic chambers. The parameters estimated for each specimen of each species were O_2_ consumption (mg/g/h) and wet weight (g). Specimens of both species were subjected to experimental conditions simulating the climate change scenarios detailed above.

The dataset is available via the MedOBIS (Mediterranean node of Ocean Biodiversity Information System) Integrated Publishing Toolkit (IPT) which has been established through the LifeWatchGreece Research Infrastructure and is hosted in the Institute of Marine Biology, Biotechnology and Aquaculture (IMBBC) of the Hellenic Centre for Marine Research (HCMR). The data are also harvested by and made available through the Ocean Biodiversity Information System (OBIS) and through the Global Biodiversity Information Facility (GBIF). The dataset is available as a DarwinCore Archive and all fields are mapped according to DarwinCore terms.

The current publication refers to the "extended measurement or fact" source file (txt file) that is associated with the particular dataset. Additional details about the sampling events and the samples can be found in the "event" and "occurence" source files respectively (txt files) associated with the same dataset. This publication refers to the most recent version of the dataset available through the IPT server or MedOBIS. Future changes to the dataset due to quality control activities might change its content or structure.

**Data set 1.** 

Column label	Column description
id	A unique identifier for the record within the dataset or collection, autoincrementingnumber automatically added by the system (same with eventID).
institutionCode	The name (or acronym) in use by the institution having custody of the object(s) or information referred to in the record.
datasetName	The name identifying the data set from which the record was derived.
eventID	An identifier for the set of information associated with a dwc:Event (something that occurs at a place and time).
parentEventID	An identifier for the broader dwc:Event that groups this and potentially other dwc:Events.
eventType	The nature of the dwc:Event.
eventDate	The date-time or interval during which a dwc:Event occurred.
year	The four-digit year in which the dwc:Event occurred, according to the Common Era Calendar.
month	The integer month in which the dwc:Event occurred.
day	The integer day of the month on which the dwc:Event occurred.
samplingProtocol	The names of, references to, or descriptions of the methods or protocols used during a dwc:Event.
locationID	An identifier for the set of dcterms:Location information. May be a global unique identifier or an identifier specific to the data set.
country	The name of the country or major administrative unit in which the dcterms:Location occurs.
countryCode	The standard code for the country in which the dcterms:Location occurs.
locality	The specific description of the place.
verbatimLocality	The original textual description of the place.
minimumDepthInMeters	The lesser depth of a range of depth below the local surface, in meters.
maximumDepthInMeters	The greater depth of a range of depth below the local surface, in meters.
decimalLatitude	The geographic latitude (in decimal degrees, using the spatial reference system given in dwc:geodeticDatum) of the geographic center of a dcterms:Location.
decimalLongitude	The geographic longitude (in decimal degrees, using the spatial reference system given in dwc:geodeticDatum) of the geographic center of a dcterms:Location.
geodeticDatum	The ellipsoid, geodetic datum, or spatial reference system (SRS) upon which the geographic coordinates given in dwc:decimalLatitude and dwc:decimalLongitude are based.
coordinateUncertaintyInMeters	The horizontal distance (in meters) from the given dwc:decimalLatitude and dwc:decimalLongitude describing the smallest circle containing the whole of the dcterms:Location.
georeferenceProtocol	A description or reference to the methods used to determine the spatial footprint, coordinates, and uncertainties.
OccurrenceID	An identifier for the dwc:Occurrence (as opposed to a particular digital record of the dwc:Occurrence).
measurementType	Τhe nature of the measurement, fact, characteristic, or assertion. For the present study the measurement types were temperature, pH, oxygen consumption rate and wet weight.
measurementValue	The value of the measurement, fact, characteristic, or assertion.
measurementUnit	The units associated with the dwc:measurementValue.
basisOfRecord	The specific nature of the data record.
individualCount	The number of individuals present at the time of the dwc:Occurrence.
OccurrenceStatus	A statement about the presence or absence of a dwc:Taxon at a dcterms:Location.
occurrenceRemarks	Comments or notes about the dwc:Occurrence.
IdentifiedBy	A list (concatenated and separated) of names of people, groups, or organizations who assigned the dwc:Taxon to the subject.
IdentificationVerificationStatus	A categorical indicator of the extent to which the taxonomic identification has been verified to be correct.
ScientificNameID	An identifier for the nomenclatural (not taxonomic) details of a scientific name.
scientificName	The full scientific name, with authorship and date information if known.
ScientificNameAuthorship	The authorship information for the dwc:scientificName formatted according to the conventions of the applicable dwc:nomenclaturalCode.

## Additional information

### Results - data analysis

**Figure 4.**
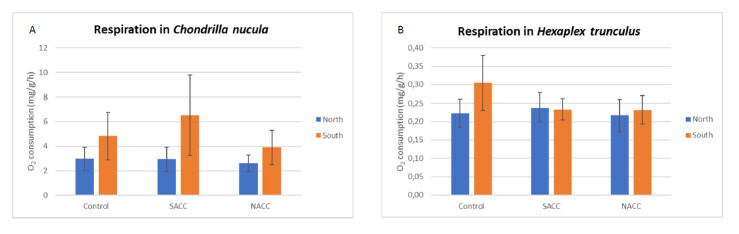
Graphical representation of the respiration of A) *Chondrilla nucula* and B) *Hexaplex trunculus* measured as oxygen consumption rate (mg O_2_/g/h) for specimens from the two populations (North and South) maintained under the three experimental treatments (Control, South Aegean Climate Change - SACC, North Aegean Climate Change - NACC). Error bars represent standard error (±SE).

The average values of the oxygen consumption rates (mg O_2_/g/h) and wet weight (g) estimated for the specimens of the sponge *Chondrilla nucula* and the gastropod *Hexaplex trunculus* from the two populations (north and south) and the three treatments Control (current conditions), South Aegean Climate Change (SACC) and North Aegean Climate Change (NACC) are presented in Table 1 and Fig. 4.

*Chondrilla nucula* specimens originating from the south population exhibited higher mean oxygen consumption rates in all treatments compared to those from the north population, with the most pronounced differences observed under the SACC treatment (Fig 4). More specifically, the respiration rate of *C. nucula* from the south population was higher under the SACC scenario in comparison to the control and lower than the control under the NACC scenario (Fig. 4). In contrast, specimens from the north population showed minimal variation in respiration rates across the different treatments.

A very interesting observation is the fact that sponges (3-6 mg/g/h) had an almost 20 times higher oxygen consumption rate in comparison to gastropods (0.2-0.3 mg/g/h). In *Hexaplex trunculus*, mean respiration rates for both populations were similar under the SACC and NACC treatments. However, more pronounced differences were observed between the two populations in the control treatment, with the south population showing higher respiration rates compared to the north population (Fig 4).

### Conclusions

The present study documents the impact of climate change and ocean acidification on a sessile and a semi-motile organism, the sponge *Chondrilla nucula* and the gastropod *Hexaplex trunculus* originating from different geographical populations. The experimental scenarios combined different regimes of increased temperature and low pH following the "high GHG emissions" RCP 8.5 scenario. Across all treatments, *C. nucula* exhibited substantially higher mass-specific oxygen consumption rates than *H. trunculus*, with values approximately 20 times greater. This is in agreement with fundamental physiological differences between sessile filter-feeding sponges and mobile gastropods (Hentschel et al. 2012, Riisgård et al. 1993).

For *Chondrilla nucula*, the higher respiration rates observed in the south population, particularly under the SACC scenario, may reflect greater metabolic plasticity or adaptation to warmer, more variable conditions in the southern Aegean. The northern's population more stable respiration rate across treatments may indicate a more conservative metabolic strategy under changing climate conditions (Somero 2010).

In contrast, *Hexaplex trunculus* showed relatively similar respiration rates across treatments, suggesting a lower sensitivity to the experimental conditions, although population differences in the Control scenario indicated inherent baseline physiological differences between the north and south populations. Overall, these patterns highlight the potential for geographic variation in metabolic responses, which may influence resilience and adaptation of marine species to climate change scenarios.

## Figures and Tables

**Table 1.** Average values of O_2_ consumption (mg/g/h) (±SE) and wet weight (g) measured for the two invertebrate species, *Chondrilla nucula* and *Hexaplex trunculus*, from the two populations (North and South). The three treatments were the Control (E1, current conditions), the South Aegean Climate Change (E2, SACC) and the North Aegean Climate Change (E3, NACC) scenarios. Sample size and range of wet weight (g) per treatment and population are also indicated.

**Species**	**Treatment**	**Origin**	**Sample size**	**O_2_ consumption (mg/g/h)**	**Wet weight (g)**
*Chondrilla nucula*	E1	North	8	2.97 (±0.96)	0.31 (0.14-0.48)
*Chondrilla nucula*	E1	South	3	4.83 (±1.95)	0.20 (0.12-0.34)
*Chondrilla nucula*	E2	North	16	2.94 (±0.99)	0.39 (0.09-0.70)
*Chondrilla nucula*	E2	South	3	6.50 (±3.26)	0.24 (0.12-0.38)
*Chondrilla nucula*	E3	North	12	2.60 (±0.66)	0.33 (0.20-0.56)
*Chondrilla nucula*	E3	South	14	3.91 (±1.39)	0.30 (0.08-0.52)
					
**Species**	**Treatment**	**Origin**	**Sample size**	**O_2_ consumption (mg/g/h)**	**Wet weight (g)**
*Hexaplex trunculus*	E1	North	12	0.22 (±0.04)	2.21 (1.63-3.03)
*Hexaplex trunculus*	E1	South	14	0.30 (±0.07)	2.33 (1.24-3.52)
*Hexaplex trunculus*	E2	North	12	0.24 (±0.04)	3.88 (2.69-5.67)
*Hexaplex trunculus*	E2	South	22	0.23 (±0.03)	4.30 (3.15-7.17)
*Hexaplex trunculus*	E3	North	10	0.22 (±0.04)	3.95 (2.54-5.76)
*Hexaplex trunculus*	E3	South	12	0.23 (±0.04)	4.23 (2.97-5.35)
